# Patients’ Perspectives on the Acceptability and Effectiveness of a Community Health Worker-Led Intervention to Increase Chronic Kidney Disease Knowledge and Screening among Underserved Latine Adults: The CARE 2.0 Study

**DOI:** 10.3390/bs14090750

**Published:** 2024-08-27

**Authors:** Janet Diaz-Martinez, Ivan Delgado-Enciso, Carlos Duran, Laura Kallus, Aydeivis Jean-Pierre, Brenda Lopez, Jessica Mancilla, Yoel Madruga, Gustavo A. Hernandez-Fuentes, Wayne Kotzker, Osiris Delgado-Enciso, Eric Wagner, Michelle Hospital

**Affiliations:** 1 Research Center in a Minority Institution, Florida International University (FIU-RCMI), Miami, FL 33199, USA; wagnere@fiu.edu (E.W.); hospitam@fiu.edu (M.H.); 2 Robert Stempel College of Public Health and School of Social Work, Florida International University, Miami, FL 33199, USA; ivan_delgado_enciso@ucol.mx; 3 School of Medicine, Colima University, Colima 28040, Mexico; gahfuentes@gmail.com (G.A.H.-F.); 1933osiris@gmail.com (O.D.-E.); 4 State Cancerology Institute of Colima, Health Services of the Mexican Social Security Institute for Welfare (IMSS-BIENESTAR), Colima 28085, Mexico; 5 Florida Kidney Physicians, Boca Raton, FL 33431, USA; cmartinez@flkidney.com (C.D.); wkotzker@flkidney.com (W.K.); 6 Caridad Center, Boynton Beach, FL 33472, USA; lkallus@caridad.org (L.K.); ajeanpierre@caridad.org (A.J.-P.); blopez@caridad.org (B.L.); jmancilla@caridad.org (J.M.); ymadrugareyes@caridad.org (Y.M.)

**Keywords:** chronic kidney disease, community health workers, CKD awareness, CKD screening and monitoring, intention–behavior, Hispanic, culturally tailored patient education materials, community-engaged research, acceptability, effectiveness

## Abstract

In the United States, Chronic Kidney Disease (CKD) affects approximately 1 in 7 adults. Despite its significant impact, CKD awareness, education, and screening are often lacking among underserved Latine populations, leading to poorer health outcomes and higher mortality rates. Various studies highlight the crucial role of Community Health Workers (CHWs) in improving health outcomes within minority communities both domestically and globally. However, there remains a gap in research on the acceptance and effectiveness of CHW-led interventions targeting CKD. This prospective intervention study employed a pre-post quasi-experimental design to evaluate a CHW-led educational program aimed at enhancing CKD knowledge, screening, and monitoring among Latines with low health literacy and English proficiency. CHWs utilized a culturally tailored CKD Flipchart, and 100 underserved patients received the intervention. Feedback from 85 participants who completed post-intervention surveys indicated high satisfaction with the program’s relevance and the professionalism of the CHWs. Importantly, 85% expressed a positive intention to seek kidney care following the intervention. Preliminary analysis of medical records before and after the intervention showed improvements in glycemic control (median change = −18.0, *p* = 0.014) and triglyceride levels (median change = −29.0, *p* = 0.035), suggesting the program’s effectiveness in managing CKD risk factors. These findings highlight the potential of CHW-led interventions to reduce kidney health disparities among underserved communities.

## 1. Introduction

In the United States (U.S.), Chronic Kidney Disease (CKD) affects approximately 37 million adults—1 in every 7 adults—and an additional 20 million have risk factors for its development [1,2]. Despite patient awareness and education being recognized as essential components and priorities of CKD prevention and management, supported by international guidelines and organizations [3,4], approximately 90% of patients with CKD are unaware of their condition [1,5,6]. Medically underserved and vulnerable populations bear a disproportionate burden of CKD and experience worse outcomes, including higher mortality [7,8].

Latine individuals represent the U.S.’s largest ethnic/racial minority group, with approximately 14% of Latine adults having CKD at rates paralleling those found in the general population [9,10,11]. Compounding this risk is the fact that Latine individuals have a faster progression to kidney failure—they are 1.3 times more likely to develop kidney failure compared to non-Latine White individuals [9,11]. This disparity is likely attributable to socioeconomic factors, including education, language, and a lack of health insurance [12,13,14,15,16]. The lack of recognition and education about CKD leaves Latine patients, families, and the healthcare system unaware of a substantial future risk of poor outcomes, including cardiovascular events, the need for urgent dialysis, and decreased quality of life [17,18,19,20,21,22,23].

Therefore, education is a necessary component of a comprehensive strategy to improve kidney health outcomes in Latine communities, aiming to increase awareness, enhance prevention, and promote the control of known risk factors [24,25,26,27,28,29]. Ensuring that Latine patients receive culturally and linguistically appropriate educational support is crucial for establishing greater social equity in CKD [30,31,32,33]. Additionally, it is important to note that Latine individuals are a complex and heterogeneous ethnic group, comprising individuals from various cultures, racial/genetic backgrounds, socioeconomic levels, and countries of origin.

In the United States, the National Kidney Foundation and numerous authors have acknowledged the crucial role of Community Health Workers (CHWs) in identifying, preventing, and managing the risks associated with CKD and ultimately improving outcomes for all affected individuals [34]. For instance, initiatives such as “Promotoras de Salud” have demonstrated effectiveness in improving blood pressure and fostering adherence to healthcare [35]. Studies investigating CHW-led interventions targeting diabetes management have reported reductions in depressive symptoms, enhancements in diabetes social support, and improvements in understanding diabetes self-management [36]. Furthermore, these interventions have been associated with increased engagement in type 2 diabetes self-care activities and reductions in hemoglobin A1C levels [37]. Another noteworthy intervention, the “Familias Sanas, in Spanish” program, involves health promoters educating Latino families about CKD, focusing on prevention and the management of risk factors such as diabetes and hypertension [38]. Moreover, among Latinos with poorly controlled diabetes, a 1-year community health worker intervention was associated with improvements in self-reported access to care [39].

Likewise, in Indigenous communities in Australia, a similar model of CHWs, known as Indigenous Health Workers (IHWs), has been implemented. These workers have played a crucial role in managing CKD by educating communities about the disease and facilitating access to tests and treatments [40]. CHWs have been instrumental in reducing the prevalence of CKD in these communities by improving education and prevention [41,42]. In India, community health workers have been used to combat the high incidence of CKD related to diabetes and hypertension. Programs like “The Jan Arogya Abhiyan” have integrated these workers into primary healthcare to provide education and support in managing CKD [41]. This approach to increasing awareness and disease monitoring has improved adherence to treatment regimens and reduced complications associated with CKD [42].

Despite the growing body of evidence supporting the use of CHW programs in CKD and diabetes [43,44,45,46,47,48], there remains a dearth of research investigating the acceptability and effectiveness of CHW-led interventions in addressing CKD unawareness and lack of screening for CKD [49,50,51], particularly in underserved Latine communities. To bridge this gap, this study assessed the acceptability and effectiveness of a CHW-led education intervention targeting underserved Latine individuals aimed at increasing (a) CKD knowledge and (b) CKD screening/monitoring. The intervention utilized a culturally and linguistically appropriate flipchart co-created by patients and CHWs. This research provides a window into how CHW-led interventions are perceived and experienced by Latine CKD patients.

## 2. Materials and Methods

### 2.1. Study Design

This prospective intervention study, employing a pre-post quasi-experimental design, evaluates the acceptability and early impact of a CHW-led educational intervention. We recruited 100 Latine patients diagnosed with type 2 diabetes (T2D) and hypertension who are at risk of or have Chronic Kidney Disease (CKD) and who received care at the largest free healthcare clinic in Florida. The clinic offers comprehensive services—including free medical, vision, dental, social, nutrition, and mental healthcare—to uninsured individuals living in poverty in Palm Beach County. It also features a specialized CKD clinic with volunteer nephrologists, hypertension specialists, nurse practitioners, renal dietitians, social workers, and trained CHWs. Additionally, the health education department provides periodic one-on-one and group workshops for patients with diabetes, hypertension, cardiovascular disease, and CKD.

### 2.2. Intervention Development

In the first phase of our study (CARE 1.0), we effectively integrated Community-Engaged Research principles with a Human-Centered Design approach to co-create CKD-patient education materials in Spanish [43]. This partnership involved the participation of Latine patients and community health workers (CHWs) from different Latine subgroups and resulted in the development of various CKD education prototypes, including a flipchart intended for use in one-on-one education delivered by CHWs. In the second phase (CARE 2.0), we conducted a pilot education intervention aimed at increasing CKD knowledge, screening, and monitoring among Latine patients with low health literacy and English proficiency and living in under-resourced communities using this culturally tailored CKD flipchart and evaluated the patients’ perspectives on the acceptability and effectiveness of this intervention. The co-created flipchart addressed CKD topics such as definition, diagnosis, lab interpretation, prevention, management, lifestyle modifications, medication adherence, and navigating the healthcare system in the community-based free clinic. Participants also received a CKD handout summarizing the educational content, including a graphic of CKD stages, disease progression risks based on eGFR and urine albuminuria, and therapeutic targets for diabetes and blood pressure control to prevent or delay CKD progression.

The educational intervention aimed to raise awareness about Chronic Kidney Disease (CKD) among patients with diabetes and hypertension and to promote regular CKD screening and monitoring to prevent disease progression. This intervention was built upon findings from the CARE 1.0 study [43], which identified barriers such as fear, low-risk perception, and poor understanding of CKD. To address these issues, the intervention sought to improve CKD knowledge in a culturally relevant manner, helping patients understand CKD stages, interpret lab results, and access appropriate medical and nutritional care. Additionally, it connected uninsured patients with free medical, social, nutritional, and educational services provided by the clinic’s CKD program. Delivered by trained CHWs using the CKD-Flipchart, the sessions lasted 45 min to 1 h, depending on individual needs, and were conducted in Spanish to ensure accessibility.

### 2.3. Training of CHWs

Twelve Latine CHWs from diverse communities (Mexico, Colombia, Peru, Puerto Rico, Cuba, Venezuela, and Uruguay) underwent 20 h of training.

The curriculum included CKD flipchart content, effective communication strategies using motivational interviewing techniques [52], cultural competence, and navigation of free CKD clinic services and community resources. The training also covered how to assist patients with arranging medical appointments, laboratory tests, transportation, accessing CKD educational resources, food and nutrition services, and navigating free clinic services in. general. Additionally, CHWs were instructed in basic research methodology, including survey administration for post-intervention assessment. They collaborated with the research team to develop and iteratively review surveys, aiming to evaluate CKD knowledge and the acceptability of the intervention.

### 2.4. Recruitment of Patients

Recruitment approaches included various methods to reach potential participants. Initially, the Electronic Medical Record (EMR) of the community health center site of the research study was queried to identify individuals who meet the following criteria: (a) Latine, (b) aged 18 years or older, (c) diagnosed with Type 2 Diabetes (T2D) or displaying laboratory results consistent with T2D (HbA1C > 7%), (d) medical diagnosis of hypertension, (e) eligible to receive free medical and social services, and (f) not having received any previous CKD education. Also, collaboration with community partners was utilized to target patients with T2D and hypertension. This outreach strategy was conducted by project staff, who set up tables at prominent community locations to promote the study. All participants were eligible to receive services at this community-based clinic. Eligibility requirements include being uninsured, living at or below 200% of the federal poverty level [53], and residing in Palm Beach County, Florida.

### 2.5. Assessment and Measurements of Acceptability and Effectiveness

We utilized a comprehensive approach to assess acceptability and effectiveness. Acceptability was evaluated based on a framework [54], employing self-report measures in surveys co-developed by the research team, which included members of the community and trained CHWs. For acceptability measurements, we co-developed a questionnaire comprising 9 questions based on the framework [54]. These questions included inquiries such as the perceived usefulness of the education on kidney disease, satisfaction with the support received by CHWs, and intentions to recommend the education to others, among others. Responses were rated on a scale from 1 to 5, with varying degrees of agreement or usefulness.

To gauge effectiveness, we compared baseline and post-intervention CKD self-reported knowledge and participants’ behavioral intention [55] toward kidney healthcare, specifically the likelihood of attending medical appointments with primary doctors or kidney specialists for CKD screening and disease monitoring after receiving the CHW-led education. CKD knowledge was assessed indirectly; instead of a formal test using scores, we asked participants if they felt their knowledge had increased due to the intervention. This approach provided insights into the perceived impact of the educational materials and the CHW-led education, though it did not quantify actual CKD knowledge score changes. We used a scale where participants rated their knowledge from 1 (“I do not know anything”) to 4 (“I know a lot”) for both the pre- and post-intervention assessments. This methodology is commonly used when direct measurement of knowledge change is not feasible, and subjective perceptions of learning and impact are valued in such evaluations [55,56,57,58,59,60].

Additionally, we implemented a questionnaire to indicate participants’ behavioral intention [55] toward kidney healthcare, specifically the likelihood of seeking medical appointments with primary doctors or kidney specialists for CKD screening and disease monitoring after receiving CHW-led education. Responses ranged from “extremely unlikely” to “extremely likely” on a scale from 1 to 5.

All surveys for acceptability and effectiveness (indirect CKD knowledge assessment, behavioral intention) were co-developed in collaboration with the community and underwent iterative reviews until the final version was refined. Given the low health and numeracy literacy of the patient population, the research team leveraged previous experiences with community-engaged methodologies, including insights from the CARE 1.0 study and the formation of a CKD advisory board [43]. Phone surveys were administered within three weeks post-intervention by a CHW who was not involved in delivering the intervention. For exploratory purposes, we collected preliminary data from electronic medical records (EMR) to assess the early impact of the intervention on biochemical parameters. These data included blood pressure, glycemic control, estimated Glomerular Filtration Rate (eGFR), and urinary albumin-to-creatinine ratio (uACR), which were compared before and after the intervention. All EMR data were collected within the three-month window both before and after the intervention.

### 2.6. Statistical Analysis

The statistical analysis encompassed several methods to comprehensively assess the data. The Kolmogorov–Smirnov test was initially utilized to ascertain the normality of the quantitative data distribution, while Levene’s test was employed to ensure the equality of variances. Quantitative variables with a normal distribution were presented as the mean ± standard deviation. For non-normally distributed quantitative data or ordinal data, such as Likert-type scales, the median and interquartile range (25th–75th percentile, Q1–Q3) were employed. Categorical variables were analyzed using odds ratios (OR) with 95% confidence intervals and the Fisher’s exact test. Intragroup data (before vs. after) were compared using the Wilcoxon signed-rank test, while the Mann–Whitney U test was utilized for intergroup comparisons of quantitative data with a non-normal distribution. Changes in biochemical parameter values were calculated by subtracting the post-intervention value from the pre-intervention value. Additionally, to assess the predictive capacity of various variables for a patient’s refusal to visit the doctor and undergo renal function tests, the areas under the ROC curve (AUCs) were calculated along with their 95% confidence intervals and corresponding *p*-values. All statistical analyses were conducted using SPSS software, version 20 (IBM Corp., Armonk, NY, USA) [61].

## 3. Results

**Figure 1:**
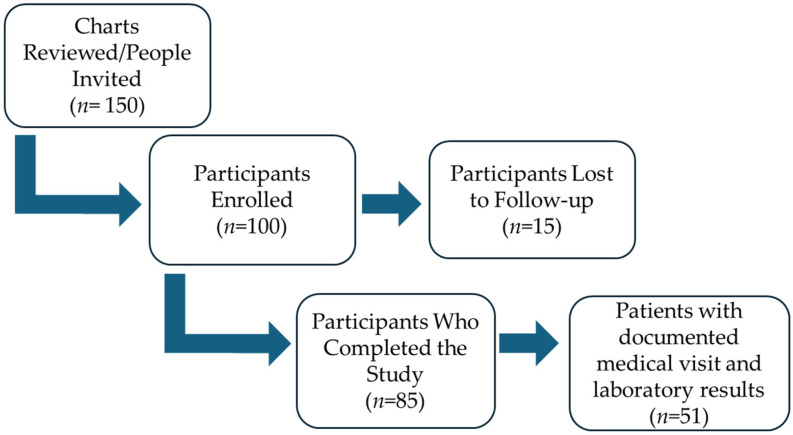
Flow chart of study participation.

Figure 1 presents a flow diagram illustrating the total number of charts reviewed, participants invited and enrolled, and the number of participants who completed the study or were lost to follow-up. This diagram provides a visual summary of the participant flow. Table 1 provides a comprehensive overview of the demographic, clinical, and socioeconomic characteristics of the sample (*n* = 100). The average age of participants was 55.6 years, with a standard deviation of 10.5 years. Notably, the majority of participants identified as female (68%) and hail from various Latin American countries, including Mexico, Guatemala, Honduras, El Salvador, Nicaragua, and Colombia. Among the sample, 82% had T2D, 75% had hypertension, and 13% had CKD, ranging from stages 1 to 4. Among the CHWs, 11 were female and 1 was male, with an average age of 52 years and a range of experience from 0 to 15 years (with a median experience of 9 years). Following the intervention, 85 out of 100 patients completed the post-intervention evaluation. Of these, 51 had laboratory work and medical visits documented in the EMR during the 3-month study period.

### 3.1. CKD Knowledge Assessment

Table 2 presents a comparison of patients’ perceptions of their knowledge levels about kidney disease topics before and after the educational intervention. Of all participants who received the education and answered post-intervention questionnaires (*n* = 85), only 7 patients were aware they had CKD.

### 3.2. Gender Differences in the Impact of Educational Intervention and Risk Perception in Kidney Disease Knowledge

Regarding the perception of “knowing quite a lot/a lot” about the 9 analyzed topics of kidney disease, it was observed that before the educational intervention, women exhibited a lower number of mastered topics (median 0, Q1–Q3 = 0–2) compared to men (median 1, Q1–Q3 = 0–3) (*p* = 0.022, Mann–Whitney U test). However, after the intervention, both women (median 9, Q1–Q3 = 7–9) and men (median 9, Q1–Q3 = 8–9) did not display differences in the number of topics they reported “knowing quite a lot/a lot” (*p* = 0.330, Mann–Whitney U test). Moreover, individuals classified as having “high risk” to “extremely high risk of kidney disease progression” or not did not demonstrate differences in the number of mastered topics, both before (median 1, Q1–Q3 = 0–2 vs. median 0, Q1–Q3 = 0–3, respectively, *p* = 0.878) and after the intervention (median 9, Q1–Q3 = 7.5–9 vs. median 9, Q1–Q3 = 7–9, *p* = 0.713).

### 3.3. Patients’ Perspectives on the Acceptability of the CHW-Led Education

Participants provided insights into the acceptability of the education intervention, focusing on various aspects such as content, perception of its usefulness, satisfaction, the CHW, and recommendations for program enhancement (Table 3). Among all participants who completed the post-survey (*n* = 85), 100% reported satisfaction with the program, with positive feedback on the usefulness and appropriateness of the covered topics, as well as the program’s structure and flow. All respondents expressed satisfaction with the CHW’s professionalism, resourcefulness, knowledge, and level of engagement, and they will recommend the education to others.

In response to an open-ended question about further education interests regarding CKD, most patients (59%) expressed a desire for additional information on topics such as (a) kidney-friendly diets, (b) self-management strategies, and (c) medications for prevention. Moreover, 20% expressed interest in understanding the interconnection between diabetes, kidney disease, and cardiovascular health. Regarding any issues, they felt were missing or could be improved on the flipchart or patient handout, more than half of the feedback highlighted the need for more detailed information on specific kidney-friendly diets and preserving kidney health. Patients also suggested adding more content to diets that can protect kidneys, such as a low-protein and a low-sodium diet, as well as providing information on preferred foods to have and foods to avoid, how much protein and carbohydrates, and recommendable cooking techniques to preserve kidney function. Additionally, 20% of patients suggested incorporating more visuals or illustrations to enhance understanding, particularly regarding the process of dialysis and kidney transplants.

### 3.4. Predictive Capacity of Various Variables for a Patient’s Behavioral Intention to Seek Kidney-HealthCare

After receiving the CHW-led intervention, 85% of the surveyed participants expressed that it is likely or extremely likely that they will visit their doctor and undergo kidney function testing, monitoring, and medical management as prescribed. To understand the factors associated with the 15% of patients who did not express intent to seek kidney healthcare, we conducted a Receiver Operating Characteristic (ROC) analysis. The analysis examined various variables, including age, annual income, education level, household size, and the number of topics perceived to be mastered after the intervention. Surprisingly, none of these variables were found to predict the likelihood of patients denying visiting their doctor and undergoing CKD screening/monitoring. Specifically, age (AUC: 0.520, 95% CI 0.353–0.803, *p* = 0.687), annual income (AUC: 0.569, 95% CI = 0.394–0.744, *p* = 0.393), education level (AUC: 0.503, 95% CI 0.349–0.657, *p* = 0.969), household size (AUC: 0.404, 95% CI 0.261–0.547, *p* = 0.238), and the number of topics perceived to be mastered after the intervention (AUC: 0.551, 95% CI 0.401–0.701, *p* = 0.529) did not serve as predictors for this behavior intent. This finding underscores the need for further research to identify the specific variables influencing patients’ unwillingness to visit their doctor post-intervention, particularly among those who felt “unlikely” or “extremely unlikely” to do so. Understanding these factors is crucial for developing targeted interventions to address barriers to healthcare access and improve patient outcomes.

In examining the relationship between patients’ negative behavior intent (“unlikely” and “extremely unlikely” to visit their doctor) post-intervention, we focused on their perception of knowledge. Interestingly, among patients who expressed no behavioral intent to undergo CKD screening and monitoring tests (15 out of 15, 100%), all reported knowing “quite a bit” or “a lot” about two specific topics: (1) goals for blood pressure and glycemia, and (2) tests to verify kidney function. In contrast, among patients who expressed a likelihood of visiting their doctor post-intervention, only 72.9% (62 out of 85 patients) reported knowing “a lot” or “quite a bit” about these topics, with a statistically significant difference (*p* = 0.014). This suggests that the absence of beneficial behavior intent may not stem from a lack of information or understanding but rather from an excessive confidence in one’s knowledge of the subject. The presence of a high-risk disease progression (eGFR between 15–29 mL/min/1.73 m^2^ and/or proteinuria > 500 mg/g creatinine) did not influence the number of topics mastered, both before (median 1, Q1–Q3 = 0–2 vs. median 0, Q1–Q3 = 0–3, respectively, *p* = 0.878) and after the intervention (median 9, Q1–Q3 = 7.5–9 vs. median 9, Q1–Q3 = 7–9, respectively, *p* = 0.713).

Furthermore, the patient’s level of education significantly influenced the number of topics perceived to be mastered by patients. Before the intervention, those with a high school education or more reported mastering a higher number of topics (median 3, Q1–Q3 = 0–7) compared to those with lower education levels (median 0, Q1–Q3 = 0–1) (*p* = 0.001). However, after the intervention, no significant differences were observed between the two groups (median 9, Q1–Q3 = 8–9 vs. median 9, Q1–Q3 = 7–9, respectively, *p* = 0.135).

### 3.5. Biochemical Parameters Analysis

For exploratory purposes, we compared preliminary biochemical and clinical data from patients’ electronic medical records (EMR) within 3 months before and after the intervention for those participants who expressed a positive intent to seek kidney healthcare as a result of the intervention. Of 72 patients who reported a positive intent to seek kidney healthcare, only 51 had documented a medical visit and laboratory results within the period analyzed. Table 4 presents the median values and corresponding quartiles (Q1 and Q3) for various biochemical parameters before and after the intervention, along with the statistical significance of the observed changes. Our analysis suggests a decrease in glycemia levels after the intervention (median change = −18.0, *p* = 0.014). Additionally, there was a notable decrease in triglyceride levels (median change = −29.0, *p* = 0.035), indicating that the intervention may have had a positive influence on specific biochemical parameters related to metabolic control and CKD risk factors, particularly glycemia and triglycerides. However, various external factors (unrelated to the intervention), such as concurrent treatments, medication adjustments, or other health-related activities, may have contributed to the observed improvement. Therefore, future studies should aim to account for these potential confounding factors to assess the specific impact of the intervention more accurately on glycemic control and other biochemical parameters. On the other hand, the intervention did not significantly impact other parameters such as HbA1c, cholesterol, eGFR, and uACR. These findings indicate that while the intervention may have contributed to improvements in specific CKD risk factors, its effects on broader kidney health and metabolic outcomes require further analysis and longer-term follow-up to fully elucidate.

## 4. Discussion

Our findings provide valuable insights into the perspectives of underserved Latine patients regarding the acceptability and impact of a CHW-led education intervention on CKD knowledge and behavioral intent toward kidney healthcare. CHW programs are recognized for their role in improving medication adherence, self-management skills, and access to healthcare services, particularly within underserved populations with chronic conditions [30,44,45]. This study’s findings highlight the positive relationship between CHWs and the communities they serve [47,48,49,50,51], emphasizing their critical role in increasing access to culturally tailored CKD education and kidney care, consistent with prior research [34,43,44,45,46,47,48,49,50,51]. Moreover, the positive impact of CHWs on enhancing CKD knowledge and fostering a positive attitude toward kidney care underscores the promise of leveraging CHWs’ roles and integrating them into future healthcare models and community health strategies.

Regarding the perception of knowledge about CKD topics, a significant improvement was observed post-intervention. Before the intervention, most participants (over 80%) had limited knowledge about kidney functions, CKD risks, and renal function tests. However, after the intervention, this outcome reversed, with over 80% of participants reporting knowing “quite a bit” or “a lot” about these topics. These results underscore the effectiveness of the co-created educational material and its implementation by CHWs in improving CKD knowledge in this vulnerable population. This improvement in knowledge is crucial, as CKD is a disease often overlooked in its early stages due to the lack of obvious symptoms, making education and awareness vital for early detection and management [62,63].

The intervention not only enhanced self-reported CKD knowledge but also fostered a positive intent to seek kidney healthcare. In the post-intervention evaluation, 85% of participants indicated that they were likely or extremely likely to visit their doctor and undergo renal function tests and CKD monitoring, and 60% of participants had CKD tests post-intervention. This positive behavioral intent addresses a critical barrier in CKD management: the lack of awareness, low perceived susceptibility to CKD, and the need for periodic screening and monitoring [3,4,64].

Our analyses also revealed that certain factors, such as age, income level, education level, and household size, did not predict the likelihood of participants visiting the doctor for renal function tests. This suggests that other barriers, possibly related to confidence in acquired knowledge or psychosocial factors, could influence this behavior intent [12,15,17,22,65]. These findings underscore the complexity of the behavioral change process and the challenges in healthcare decision-making within vulnerable communities. Education and awareness alone may not be sufficient to overcome all barriers to medical care [60].

While the intervention appears to positively influence specific biochemical parameters related to metabolic control and CKD risk factors, such as glycemia and triglycerides, it did not significantly impact other indicators of kidney and metabolic health, including HbA1c, cholesterol, eGFR, and uACR. These results should be interpreted with caution. First, the biochemical parameters were measured within a three-month post-intervention window, which may be too short to draw definitive conclusions about the intervention’s impact on clinical outcomes. Second, the absence of a control group limits our ability to account for other factors, such as lifestyle changes, access and adherence to medications, and comorbidities, that may explain the variability in response [11]. These limitations highlight the need for additional studies with long-term follow-up to fully evaluate the intervention’s effects on overall kidney health and risk factors.

Participant feedback emphasized the importance of ongoing education and the need for additional information, particularly on diet and other aspects of kidney disease self-management. Patients suggested that the program could benefit from more detailed dietary recommendations tailored to different stages of kidney disease and practical strategies for preserving kidney health. This feedback is critical for continuously adapting and improving educational interventions, ensuring they remain relevant and beneficial for patients [35,54,64,66].

The high level of acceptance and satisfaction reported by participants suggests that CHWs are valued in CKD education and community support. While this study did not include a control group to make direct comparisons, the positive feedback indicates that CHWs were likely effective in engaging the underserved community on kidney health topics. This is consistent with other research showing that CHW programs can be effective in improving community health outcomes [67,68,69,70]. For example, similar programs have been successful in areas such as reducing emergency department visits [62], increasing cancer screening referrals [63], and managing chronic conditions like diabetes and hypertension [64,65]. Expanding CHW-led initiatives to other vulnerable populations might similarly enhance CKD recognition and management while reducing health disparities [35,44,48,49,50]. CHWs are particularly effective in bridging gaps between healthcare services and patients, addressing cultural and linguistic barriers that could otherwise impede access to care [24,30].

Our findings align with studies showing that CHWs can enhance disease-specific knowledge, positively impact chronic disease management, and improve access to care [35,40,41,42,49,60]. CHWs can also be integrated with other approaches, such as telemedicine, which faces challenges like technology access and acceptance [67]. For example, the combination of CHWs and telemedicine in Canada for diabetes management [68] highlights how CHWs can complement other methods to improve healthcare accessibility [69].

While our intervention did not aim to assess direct changes in CKD-related clinical outcomes as primary outcomes, the exploratory analysis of improved specific biochemical parameters—interpreted with caution—along with high participant satisfaction, increased self-reported CKD knowledge, and positive intent to seek care suggests that CHWs can effectively engage communities with low CKD awareness and serve as agents of change. Additionally, the positive feedback on culturally tailored educational materials co-created by CHWs highlights their effectiveness in addressing CKD knowledge gaps. Replicating such CHW-led programs in other vulnerable communities could further enhance CKD awareness, improve access to effective education, and facilitate CKD screening and monitoring, particularly for individuals with low health literacy and limited English proficiency living in under-resourced areas.

This study has several limitations due to its quasi-experimental design, which lacks a control group and restricts our ability to definitively attribute observed changes solely to the intervention. Although significant changes in biochemical parameters were noted, these results should be interpreted with caution due to potential influences from comorbidities, medication adherence, and lifestyle changes. Future studies should include a control group and additional sham training to better isolate the effects of CHW education and account for other variables that may impact the results [53,54]. Another limitation of this study is the absence of data on whether participants had a primary care doctor or health insurance, factors that could significantly influence their willingness and ability to seek medical care. Additionally, while most participants were recruited from a free clinic, our broader recruitment did not adequately account for varying levels of healthcare access among participants. Another limitation is the lack of information regarding the documentation status of participants, which could have provided critical insights into the role of immigration status in healthcare-seeking behavior. Finally, while immigration fears were indirectly addressed, a more direct assessment of how these fears impact the willingness to engage in medical care would have strengthened our analysis.

However, important challenges remain, such as the lack of early detection and the need to strengthen the doctor–patient relationship. Improving communication and providing clear instructions from primary care are essential not only for patients with chronic kidney disease but also for vulnerable groups more broadly. The findings highlight the effectiveness of using community health workers (CHWs) as a strategy that not only facilitates interventions but also stands out for its ability to build trust and establish long-term relationships with patients. Compared to other strategies, such as chronic disease management programs utilizing telemedicine or technology-based interventions, CHWs offer a particular advantage by focusing on personal relationships, a critical element for the success of community health interventions. Further research is needed to optimize this approach, particularly in populations with significant barriers to healthcare access.

One limitation of this study is the absence of data on whether participants had a primary care doctor or health insurance, factors that could significantly influence their willingness and ability to seek medical care. Additionally, while most participants were recruited from a free clinic, our broader recruitment did not adequately account for varying levels of healthcare access among participants. Another key limitation is the lack of information regarding participants’ documentation status, which could have provided critical insights into the role of immigration status in healthcare-seeking behavior. Although immigration fears were indirectly addressed, a more direct assessment of how these concerns impact the willingness to engage in medical care would have strengthened our analysis. The decision not to collect data on participants’ documentation status was influenced by ethical considerations. While this information is rarely gathered in health research due to concerns about privacy breaches, discomfort, and the potential for a “chilling effect” on participant trust [71,72,73,74], it is important to understand the barriers faced by immigrants. While our educational approach, which involved co-creating CKD materials with CHWs and patients and having a CHW conduct the intervention, indirectly addressed CKD care-related fears and trust issues with the educational content, a more direct assessment of these factors would have strengthened our analysis. Future research should address these gaps to better understand the barriers and facilitators to healthcare for vulnerable Latine populations.

## 5. Conclusions

Our study underscores the high acceptability of CHW-led interventions and culturally tailored educational materials, highlighting their positive impact on enhancing CKD knowledge and fostering positive behavioral intent toward kidney healthcare among the Latine population with or at risk for CKD. Integrating CHWs into clinical care teams is a promising strategy for delivering culturally appropriate CKD education and improving kidney health outcomes. Additionally, establishing sustainable funding mechanisms is crucial for scaling and maintaining CHW programs in safety-net settings.

## Figures and Tables

**Table 1:** Characteristics of patient participants.

Covariate	All (*n* = 100)
Age (years)–Mean (SD)	55.6 ± 10.5
Female	68.0%
Obesity	33.9%
Country of Origin	
Mexico	43.0%
Guatemala	19%
Honduras	9.0%
El Salvador	5.0%
Nicaragua	4.0%
Colombia	4.0%
Other Latin countries	16.0%
Former smoker	19%
Alcohol use	
None	82.0%
Occasional	16.0%
Moderate	1.0%
Heavy	1.0%
Medical Diagnosis	
Obesity	27.0%
Hypertension	75.0%
Diabetic	82.0%
Hyperlipidemia	52.0%
Cardiopathy	9.0%
Hypothyroidism	10.0%
CKD (1–4)	13.0%
Cancer	5.0%
Depression	9.0%
CKD (high risk of disease progression) *	36.0%
Literate	90.0%
Semi-literate	10%
Home	
Single-level house	43.0%
Apartment	26.0%
Trailer	22.0%
Multi-level house	1.0%
Other	8.0%
Household members–Mean (SD)	2.0 ± 1.1
Education	
Never attended school	8.0%
Less than a high school degree	73.0%
High school diploma/GED	6.0%
More than high school	13.0%
Unemployed	53.0%
Income/year–Mean (SD)	$17,993.2 ± $9955.4

* CKD (high risk to extremely high risk of disease progression) based on CKD Heat Map categories: Low or no risk: eGFR > 60 mL/min/1.73 m^2^ and proteinuria < 150 mg/g creatinine. Moderately increased risk: eGFR between 30 and 59 mL/min/1.73m^2^ and/or between 150 and 500 mg/g creatinine. High risk: eGFR between 15–29 mL/min/1.73 m^2^ and/or proteinuria > 500 mg/g creatinine. Very high risk: eGFR < 15 mL/min/1.73 m^2^, regardless of the level of proteinuria. Extremely high risk: This category includes individuals with end-stage kidney disease (ESKD) requiring renal replacement therapy such as dialysis or kidney transplantation.

**Table 2:** Patient’s perception of their knowledge level regarding kidney disease topics before and after the educational intervention.

Knowledge about the Topic of:		Nothing/Little	A Lot	OR **	IC95%		*p* ***
Kidney Functions	Before	79.0%	21.0%	25.2	11.8	53.6	<0.001
	After	12.0%	88.0%				
What is Kidney Disease	Before	79.0%	21.0%	25.2	11.8	53.6	<0.001
	After	13.0%	87.0%				
Who is at Risk	Before	64.0%	36.0%	23.6	9.9	56.4	<0.001
	After	7.0%	93.0%				
Tests for Kidney Function	Before	84.0%	16.0%	29.7	13.8	64.0	<0.001
	After	15.0%	85.0%				
Progression/Visit Nephrologist	Before	87.0%	13.0%	32.6	14.9	71.4	<0.001
	After	17.0%	83.0%				
Diet and Kidney Disease	Before	84.0%	16.0%	29.7	13.8	64.0	<0.001
	After	15.0%	85.0%				
Therapeutic Goals to Protect Kidneys *	Before	77.0%	23.0%	17.6	8.6	35.7	<0.001
	After	16.0%	84.0%				
Medicines for Kidney and Heart	Before	91.0%	9.0%	27.3	12.1	61.7	<0.001
	After	27.0%	73.0%				
Therapies available if Kidney Function Worsens	Before	83.0%	17.0%	20.814	10.1	42.9	<0.001
	After	19.0%	81.0%				

* Your blood pressure and glucose level goal to protect your kidneys ** Crude Odds ratio were performed with before education data set as the reference group. *** Fisher’s exact test.

**Table 3:** Acceptability of the education intervention.

	Median	Q1	Q3
1. How useful did you find the education about kidney disease? *	4	4	5
2. I learned new things about kidney disease **	5	4	5
3. The CHWs were well-informed and friendly **	5	5	5
4. I felt comfortable discussing my concerns about kidney disease **	5	5	5
5. The words and vocabulary used in the flipchart were easy for me to understand **	5	5	5
6. The images/photos used in the flipchart helped me understand kidney disease **	5	5	5
7. Overall, I was satisfied with the support and guidance provided during the education **	5	5	5
8. The education received will help me with my kidney health and making lifestyle changes **	5	5	5
9. I will recommend this education to others **	5	5	5

* 1: Not useful; 2: Slightly useful; 3: Moderately useful; 4: Very useful; 5: Extremely useful. ** 1: Strongly disagree; 2: Disagree; 3: Neither agree nor disagree; 4: Agree; 5: Strongly agree.

**Table 4:** Comparison of biochemical parameters three months before and after CKD education intervention for those participants that reported positive behavior intent to seek kidney healthcare **.

Variable	Before			After			*p* *	Change		
	Median	Q1	Q3	Median	Q1	Q3		Median	Q1	Q3
Glycemia	151.0	118.5	201.8	130.0	109.0	167.0	0.014	−18.0	−52.3	7.3
HbA1c	7.4	6.5	8.1	7.4	6.8	8.5	0.509	0.2	−0.7	0.9
Chol-LDL	75.0	56.0	113.0	71.0	48.0	104.0	0.284	−2.0	−31.0	13.0
Chol-HDL	47.0	40.0	55.0	47.0	38.0	53.0	0.791	0.0	−4.0	4.0
Trig.	149.0	109.0	201.5	140.0	107.0	211.0	0.035	−29.0	−85.8	30.5
eGFR	94.0	65.3	107.0	85.0	60.5	103.5	0.942	0.0	−6.0	5.0
uACR	22.0	9.3	97.0	30.0	12.3	217.3	0.638	−2.0	−23.0	66.0

* Wilcoxon signed-rank test, Before vs. After. Glycemia (fasting glycemia), milligrams per deciliter (mg/dL). HbA1c (Hemoglobin A1c), percentage (%). Chol-LDL (low-density lipoprotein cholesterol), milligrams per deciliter (mg/dL). Chol-HDL (high-density lipoprotein cholesterol), milligrams per deciliter (mg/dL). Trig. (Triglycerides), milligrams per deciliter (mg/dL). eGFR (Estimated Glomerular Filtration Rate), milliliters per minute per 1.73 square meters (mL/min/1.73 m^2^). uACR (Urinary Albumin-to-Creatinine Ratio), milligrams per gram (mg/g). Change (value after education minus value before education). ** *n* = 51 (patients with laboratory work and medical visits documented in the EMR during the 3-month study period).

## Data Availability

The data presented in this study are available on request from the corresponding author. The data are not publicly available, as we only asked for informed consent from the participants to share data with other researchers on request.
